# 

**Published:** 1874-09

**Authors:** 


### MAD RIVER VALLEY DENTAL ASSOCIATION.

The regular semi-annual meeting of the Mad River Valley Dental Association will be held in Dayton, O., on October 6th, 1874, commencing at 10 o'clock, a. m.

SUBJECTS FOR DISCUSSION.

1st. Histology of the teeth.

2d. Diseases of the gums, and their treatment.

3d. Management of exposed pulps.

4th. What are the requisites to secure success in filling teeth?

5th. Smooth point pluggers, are they superior to serrated instruments?

6th. Removal of superficial decay, will it, in all cases, effectually arrest the disease?

7th. Best method of using the tooth brush, quality of brush and how often?

8th. Flexible rubber disks.

9th. What constitutes success in dental practice?

10th. Dental education.

A cordial invitation is extended to all to be present.

A. J. Grosvenor, Sec. S. B. Tizzard, Pres.

### IMPORTANT TO MEDICAL STUDENTS.

The Trustees of the Louisville Medical College, (Louisville, Ky.,) appreciating the impoverished condition of the whole country, have determined to grant a Beneficiary Scholarship to any young man, who, sufficiently educated to study medicine and of good character, is unable to pay for his education. To secure this valuable aid, application, with a full statement of the case, should be made without delay to Dr. E. S. Gaillard, Dean, Louisville, Ky.

